# 5-Chloro-3-cyclo­pentyl­sulfinyl-2-methyl-1-benzofuran

**DOI:** 10.1107/S1600536811019775

**Published:** 2011-06-04

**Authors:** Pil Ja Seo, Hong Dae Choi, Byeng Wha Son, Uk Lee

**Affiliations:** a Department of Chemistry, Dongeui University, San 24 Kaya-dong Busanjin-gu, Busan 614-714, Republic of Korea; b Department of Chemistry, Pukyong National University, 599-1 Daeyeon 3-dong, Nam-gu, Busan 608-737, Republic of Korea

## Abstract

In the title compound, C_14_H_15_ClO_2_S, the cyclo­pentyl ring adopts an envelope conformation. In the crystal, mol­ecules are linked through weak inter­molecular C—H⋯O hydrogen bonds. The crystal structure also exhibits a slipped π–π inter­action between the furan and benzene rings of neighbouring mol­ecules [centroid–centroid distance = 3.784 (3) Å, inter­planar distance = 3.199 (3) Å and slippage = 2.021 (3) Å].

## Related literature

For the biological activity of benzofuran compounds, see: Aslam *et al.* (2009 ▶); Galal *et al.* (2009 ▶); Khan *et al.* (2005 ▶). For natural products with benzofuran rings, see: Akgul & Anil (2003 ▶); Soekamto *et al.* (2003 ▶). For a structural study of the related compound, 5-bromo-3-cyclo­pentyl­sulfinyl-2-methyl-1-benzofuran, see: Seo *et al.* (2011 ▶).
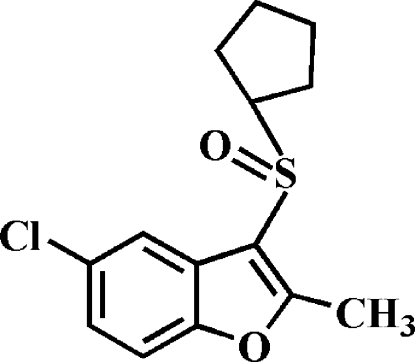


## Experimental

### 

#### Crystal data

C_14_H_15_ClO_2_S
*M* *_r_* = 282.77
Triclinic, 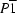

*a* = 6.3337 (3) Å
*b* = 8.9449 (4) Å
*c* = 12.1157 (5) Å
α = 73.614 (2)°
β = 78.110 (2)°
γ = 88.087 (2)°
*V* = 644.17 (5) Å^3^
*Z* = 2
Mo *K*α radiation
μ = 0.45 mm^−1^
*T* = 173 K
0.35 × 0.26 × 0.20 mm

#### Data collection

Bruker SMART APEXII CCD diffractometer
Absorption correction: multi-scan (*SADABS*; Bruker, 2009 ▶) *T*_min_ = 0.858, *T*_max_ = 0.916
11628 measured reflections
2995 independent reflections
2514 reflections with *I* > 2σ(*I*)
*R*_int_ = 0.055

#### Refinement

*R*[*F* ^2^ > 2σ(*F* ^2^)] = 0.040
*wR*(*F* ^2^) = 0.105
*S* = 1.05
2995 reflections
164 parameters
H-atom parameters constrained
Δρ_max_ = 0.43 e Å^−3^
Δρ_min_ = −0.26 e Å^−3^

### 

Data collection: *APEX2* (Bruker, 2009 ▶); cell refinement: *SAINT* (Bruker, 2009 ▶); data reduction: *SAINT*; program(s) used to solve structure: *SHELXS97* (Sheldrick, 2008 ▶); program(s) used to refine structure: *SHELXL97* (Sheldrick, 2008 ▶); molecular graphics: *ORTEP-3* (Farrugia, 1997 ▶) and *DIAMOND* (Brandenburg, 1998 ▶); software used to prepare material for publication: *SHELXL97*.

## Supplementary Material

Crystal structure: contains datablock(s) global, I. DOI: 10.1107/S1600536811019775/zl2373sup1.cif

Structure factors: contains datablock(s) I. DOI: 10.1107/S1600536811019775/zl2373Isup2.hkl

Supplementary material file. DOI: 10.1107/S1600536811019775/zl2373Isup3.cml

Additional supplementary materials:  crystallographic information; 3D view; checkCIF report

## Figures and Tables

**Table 1:** Hydrogen-bond geometry (Å, °)

*D*—H⋯*A*	*D*—H	H⋯*A*	*D*⋯*A*	*D*—H⋯*A*
C5—H5⋯O2^i^	0.95	2.61	3.349 (2)	135

Symmetry code: (i) 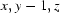.

**Fig. 1.**
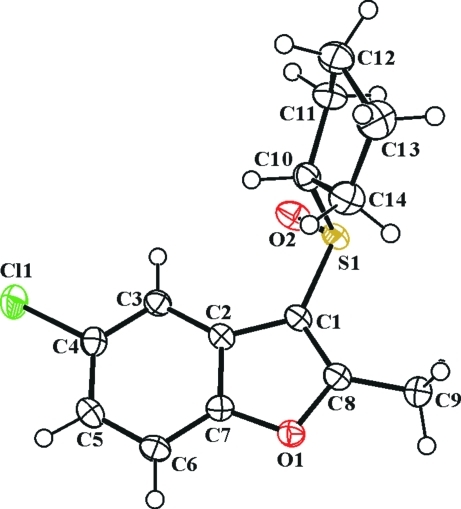
The molecular structure of the title compound with the atom numbering scheme. Displacement ellipsoids are drawn at the 50% probability level. H atoms are presented as a small spheres of arbitrary radius.

**Fig. 2.**
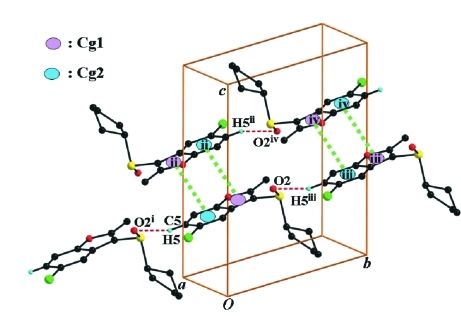
A view of the C—H···O and π–π interactions (dotted lines) in the crystal structure of the title compound. [Symmetry codes: (i) x, y - 1, z ; (ii) - x + 1, - y, - z + 1; (iii) x, y + 1, z; (iv) - x + 1, - y + 1, - z + 1.]

## supplementary crystallographic information

### Comment

Recently, many compounds involving a benzofuran ring system have drawn much attention due to their inetresting pharmacological properties such as antibacterial and antifungal, antitumor and antiviral, and antimicrobial activities (Aslam *et al.*, 2009; Galal *et al.*, 2009; Khan *et al.*, 2005). These benzofuran derivatives occur in a wide range of natural products (Akgul & Anil, 2003; Soekamto *et al.*, 2003). As part of our ongoing project of the substituent effect on the solid state structures of 3-cyclopentylsulfinyl-5-halo-2-methyl-1-benzofuran analogues (Seo *et al.*, 2011), we report herein the crystal structure of the title compound.

In the title molecule (Fig. 1), the benzofuran unit is essentially planar, with a mean deviation of 0.009 (1) Å from the least-squares plane defined by the nine constituent atoms. The cyclopentyl ring is in the envelope form. The crystal packing (Fig. 2) is stabilized by weak intermolecular C—H···O hydrogen bonds between a benzene H atom and the O atom of the sulfinyl group (Table 1; C5—H5···O2^i^). The crystal packing (Fig. 2) is further stabilized by a weak slipped π–π interaction between the furan and benzene rings of neighbouring molecules, with a Cg1···Cg2^ii^ distance of 3.784 (3) Å and an interplanar distance of 3.199 (3) Å resulting in a slippage of 2.021 (3) Å (Cg1 and Cg2 are the centroids of the C1/C2/C7/O1/C8 furan ring and the C2–C7 benzene ring, respectively).

### Experimental

77% 3-chloroperoxybenzoic acid (269 mg, 1.2 mmol) was added in small portions to a stirred solution of 5-chloro-3-cyclopentylsulfanyl-2-methy1-benzofuran (293 mg, 1.1 mmol) in dichloromethane (30 mL) at 273 K. After being stirred at room temperature for 4h, the mixture was washed with saturated sodium bicarbonate solution and the organic layer was separated, dried over anhydrous magnesium sulfate, filtered and concentrated at reduced pressure. The residue was purified by column chromatography (hexane–ethyl acetate, 1:1 v/v) to afford the title compound as a colorless solid [yield 74%, m.p. 386–387 K; *R*_f_ = 0.48 (hexane–ethyl acetate, 1:1 v/v)]. Single crystals suitable for X-ray diffraction were prepared by slow evaporation of a solution of the title compound in ethyl acetate at room temperature.

### Refinement

All H atoms were positioned geometrically and refined using a riding model, with C—H = 0.95 Å for aryl, 1.00 Å for methine, 0.99 Å for methylene and 0.98 Å for methyl H atoms, respectively.  *U*_iso_(H)  = 1.2*U*_eq_(C)  for aryl,  methine,  methylene,  and 1.5*U*_eq_(C) for methyl H atoms.

### Crystal data

**Table:**

C_14_H_15_ClO_2_S	*Z* = 2
*M**_r_* = 282.77	*F*(000) = 296
Triclinic, *P*1	*D*_x_ = 1.458 Mg m^−^^3^
Hall symbol: -P 1	Mo *K*α radiation, λ = 0.71073 Å
*a* = 6.3337 (3) Å	Cell parameters from 4102 reflections
*b* = 8.9449 (4) Å	θ = 2.4–27.6°
*c* = 12.1157 (5) Å	µ = 0.45 mm^−^^1^
α = 73.614 (2)°	*T* = 173 K
β = 78.110 (2)°	Block, colourless
γ = 88.087 (2)°	0.35 × 0.26 × 0.20 mm
*V* = 644.17 (5) Å^3^	

### Data collection

**Table:**

Bruker SMART APEXII CCD diffractometer	2995 independent reflections
Radiation source: rotating anode	2514 reflections with *I* > 2σ(*I*)
graphite multilayer	*R*_int_ = 0.055
Detector resolution: 10.0 pixels mm^-1^	θ_max_ = 27.7°, θ_min_ = 1.8°
φ and ω scans	*h* = −8→8
Absorption correction: multi-scan (*SADABS*; Bruker, 2009)	*k* = −11→11
*T*_min_ = 0.858, *T*_max_ = 0.916	*l* = −15→14
11628 measured reflections	

### Refinement

**Table:**

Refinement on *F*^2^	Primary atom site location: structure-invariant direct methods
Least-squares matrix: full	Secondary atom site location: difference Fourier map
*R*[*F*^2^ > 2σ(*F*^2^)] = 0.040	Hydrogen site location: difference Fourier map
*wR*(*F*^2^) = 0.105	H-atom parameters constrained
*S* = 1.05	*w* = 1/[σ^2^(*F*_o_^2^) + (0.0486*P*)^2^ + 0.2468*P*] where *P* = (*F*_o_^2^ + 2*F*_c_^2^)/3
2995 reflections	(Δ/σ)_max_ = 0.001
164 parameters	Δρ_max_ = 0.43 e Å^−^^3^
0 restraints	Δρ_min_ = −0.26 e Å^−^^3^

### Special details

**Table:**

Geometry. All esds (except the esd in the dihedral angle between two l.s. planes) are estimated using the full covariance matrix. The cell esds are taken into account individually in the estimation of esds in distances, angles and torsion angles; correlations between esds in cell parameters are only used when they are defined by crystal symmetry. An approximate (isotropic) treatment of cell esds is used for estimating esds involving l.s. planes.
Refinement. Refinement of F^2^ against ALL reflections. The weighted R-factor wR and goodness of fit S are based on F^2^, conventional R-factors R are based on F, with F set to zero for negative F^2^. The threshold expression of F^2^ > 2sigma(F^2^) is used only for calculating R-factors(gt) etc. and is not relevant to the choice of reflections for refinement. R-factors based on F^2^ are statistically about twice as large as those based on F, and R- factors based on ALL data will be even larger.

### Fractional atomic coordinates and isotropic or equivalent isotropic displacement parameters (Å^2^)

**Table:**

	*x*	*y*	*z*	*U*_iso_*/*U*_eq_	
Cl1	1.11290 (7)	0.07397 (6)	0.16222 (5)	0.03628 (15)	
S1	0.45480 (7)	0.51787 (5)	0.33115 (4)	0.02483 (14)	
O1	0.24300 (19)	0.08162 (14)	0.43094 (11)	0.0254 (3)	
O2	0.6818 (2)	0.54497 (15)	0.33897 (13)	0.0345 (3)	
C1	0.4123 (3)	0.3158 (2)	0.36271 (16)	0.0238 (4)	
C2	0.5584 (3)	0.2048 (2)	0.32358 (15)	0.0216 (4)	
C3	0.7684 (3)	0.2107 (2)	0.25933 (16)	0.0238 (4)	
H3	0.8512	0.3054	0.2287	0.029*	
C4	0.8508 (3)	0.0724 (2)	0.24220 (16)	0.0247 (4)	
C5	0.7343 (3)	−0.0685 (2)	0.28622 (17)	0.0267 (4)	
H5	0.7976	−0.1606	0.2714	0.032*	
C6	0.5269 (3)	−0.0747 (2)	0.35141 (17)	0.0263 (4)	
H6	0.4450	−0.1697	0.3833	0.032*	
C7	0.4445 (3)	0.0636 (2)	0.36806 (15)	0.0224 (4)	
C8	0.2289 (3)	0.2361 (2)	0.42738 (16)	0.0246 (4)	
C9	0.0257 (3)	0.2831 (2)	0.49225 (18)	0.0313 (4)	
H9A	0.0574	0.3630	0.5281	0.047*	
H9B	−0.0443	0.1921	0.5539	0.047*	
H9C	−0.0707	0.3252	0.4378	0.047*	
C10	0.4471 (3)	0.5660 (2)	0.17647 (16)	0.0252 (4)	
H10	0.5579	0.5056	0.1366	0.030*	
C11	0.4876 (3)	0.7408 (2)	0.11699 (17)	0.0305 (4)	
H11A	0.4244	0.8030	0.1710	0.037*	
H11B	0.6440	0.7665	0.0894	0.037*	
C12	0.3739 (3)	0.7690 (2)	0.01427 (18)	0.0343 (4)	
H12A	0.3433	0.8808	−0.0155	0.041*	
H12B	0.4619	0.7336	−0.0508	0.041*	
C13	0.1660 (3)	0.6720 (3)	0.0669 (2)	0.0414 (5)	
H13A	0.1129	0.6397	0.0055	0.050*	
H13B	0.0528	0.7324	0.1035	0.050*	
C14	0.2223 (3)	0.5299 (2)	0.15928 (19)	0.0335 (4)	
H14A	0.2250	0.4354	0.1319	0.040*	
H14B	0.1150	0.5126	0.2340	0.040*	

### Atomic displacement parameters (Å^2^)

**Table:**

	*U*^11^	*U*^22^	*U*^33^	*U*^12^	*U*^13^	*U*^23^
Cl1	0.0254 (2)	0.0385 (3)	0.0432 (3)	0.00585 (19)	−0.0012 (2)	−0.0135 (2)
S1	0.0279 (2)	0.0181 (2)	0.0302 (3)	0.00119 (17)	−0.00814 (18)	−0.00808 (18)
O1	0.0235 (6)	0.0213 (6)	0.0307 (7)	−0.0011 (5)	−0.0035 (5)	−0.0077 (5)
O2	0.0342 (7)	0.0262 (7)	0.0469 (9)	−0.0037 (6)	−0.0199 (6)	−0.0076 (6)
C1	0.0257 (8)	0.0194 (8)	0.0273 (10)	0.0016 (7)	−0.0076 (7)	−0.0069 (7)
C2	0.0236 (8)	0.0205 (8)	0.0230 (9)	0.0018 (6)	−0.0085 (7)	−0.0070 (7)
C3	0.0240 (8)	0.0219 (9)	0.0264 (9)	0.0000 (7)	−0.0070 (7)	−0.0068 (7)
C4	0.0217 (8)	0.0286 (9)	0.0252 (9)	0.0041 (7)	−0.0075 (7)	−0.0084 (8)
C5	0.0318 (9)	0.0228 (9)	0.0298 (10)	0.0052 (7)	−0.0114 (8)	−0.0110 (8)
C6	0.0293 (9)	0.0210 (9)	0.0297 (10)	−0.0024 (7)	−0.0082 (7)	−0.0071 (8)
C7	0.0222 (8)	0.0225 (9)	0.0226 (9)	−0.0009 (6)	−0.0063 (7)	−0.0053 (7)
C8	0.0259 (9)	0.0219 (9)	0.0268 (10)	0.0010 (7)	−0.0071 (7)	−0.0069 (7)
C9	0.0264 (9)	0.0296 (10)	0.0350 (11)	0.0027 (8)	−0.0020 (8)	−0.0079 (9)
C10	0.0249 (9)	0.0228 (9)	0.0289 (10)	0.0010 (7)	−0.0063 (7)	−0.0084 (8)
C11	0.0346 (10)	0.0234 (10)	0.0325 (11)	−0.0034 (8)	−0.0108 (8)	−0.0031 (8)
C12	0.0390 (11)	0.0325 (11)	0.0315 (11)	0.0022 (8)	−0.0120 (9)	−0.0058 (9)
C13	0.0331 (11)	0.0504 (14)	0.0406 (13)	0.0013 (9)	−0.0149 (9)	−0.0074 (11)
C14	0.0294 (10)	0.0325 (11)	0.0404 (12)	−0.0035 (8)	−0.0123 (8)	−0.0087 (9)

### Geometric parameters (Å, °)

**Table:**

Cl1—C4	1.7398 (18)	C9—H9A	0.9800
S1—O2	1.4926 (13)	C9—H9B	0.9800
S1—C1	1.7571 (17)	C9—H9C	0.9800
S1—C10	1.8113 (19)	C10—C11	1.532 (2)
O1—C8	1.371 (2)	C10—C14	1.538 (2)
O1—C7	1.375 (2)	C10—H10	1.0000
C1—C8	1.355 (3)	C11—C12	1.520 (3)
C1—C2	1.446 (2)	C11—H11A	0.9900
C2—C7	1.387 (2)	C11—H11B	0.9900
C2—C3	1.390 (2)	C12—C13	1.520 (3)
C3—C4	1.380 (2)	C12—H12A	0.9900
C3—H3	0.9500	C12—H12B	0.9900
C4—C5	1.392 (3)	C13—C14	1.525 (3)
C5—C6	1.380 (3)	C13—H13A	0.9900
C5—H5	0.9500	C13—H13B	0.9900
C6—C7	1.377 (2)	C14—H14A	0.9900
C6—H6	0.9500	C14—H14B	0.9900
C8—C9	1.478 (3)		
			
O2—S1—C1	107.59 (8)	H9A—C9—H9C	109.5
O2—S1—C10	107.13 (8)	H9B—C9—H9C	109.5
C1—S1—C10	96.85 (8)	C11—C10—C14	105.36 (15)
C8—O1—C7	106.57 (13)	C11—C10—S1	111.26 (12)
C8—C1—C2	107.35 (15)	C14—C10—S1	111.01 (13)
C8—C1—S1	125.01 (14)	C11—C10—H10	109.7
C2—C1—S1	127.63 (14)	C14—C10—H10	109.7
C7—C2—C3	119.67 (16)	S1—C10—H10	109.7
C7—C2—C1	104.65 (15)	C12—C11—C10	102.68 (15)
C3—C2—C1	135.66 (16)	C12—C11—H11A	111.2
C4—C3—C2	116.77 (16)	C10—C11—H11A	111.2
C4—C3—H3	121.6	C12—C11—H11B	111.2
C2—C3—H3	121.6	C10—C11—H11B	111.2
C3—C4—C5	123.11 (17)	H11A—C11—H11B	109.1
C3—C4—Cl1	118.46 (14)	C13—C12—C11	103.48 (17)
C5—C4—Cl1	118.43 (14)	C13—C12—H12A	111.1
C6—C5—C4	120.05 (17)	C11—C12—H12A	111.1
C6—C5—H5	120.0	C13—C12—H12B	111.1
C4—C5—H5	120.0	C11—C12—H12B	111.1
C7—C6—C5	116.80 (16)	H12A—C12—H12B	109.0
C7—C6—H6	121.6	C12—C13—C14	105.84 (16)
C5—C6—H6	121.6	C12—C13—H13A	110.6
O1—C7—C6	125.67 (16)	C14—C13—H13A	110.6
O1—C7—C2	110.74 (15)	C12—C13—H13B	110.6
C6—C7—C2	123.59 (17)	C14—C13—H13B	110.6
C1—C8—O1	110.68 (15)	H13A—C13—H13B	108.7
C1—C8—C9	132.96 (17)	C13—C14—C10	105.96 (16)
O1—C8—C9	116.36 (15)	C13—C14—H14A	110.5
C8—C9—H9A	109.5	C10—C14—H14A	110.5
C8—C9—H9B	109.5	C13—C14—H14B	110.5
H9A—C9—H9B	109.5	C10—C14—H14B	110.5
C8—C9—H9C	109.5	H14A—C14—H14B	108.7
			
O2—S1—C1—C8	140.45 (16)	C1—C2—C7—O1	−0.31 (19)
C10—S1—C1—C8	−109.10 (16)	C3—C2—C7—C6	−0.7 (3)
O2—S1—C1—C2	−41.40 (18)	C1—C2—C7—C6	−179.57 (16)
C10—S1—C1—C2	69.05 (16)	C2—C1—C8—O1	−1.41 (19)
C8—C1—C2—C7	1.03 (19)	S1—C1—C8—O1	177.05 (12)
S1—C1—C2—C7	−177.38 (13)	C2—C1—C8—C9	178.34 (19)
C8—C1—C2—C3	−177.56 (19)	S1—C1—C8—C9	−3.2 (3)
S1—C1—C2—C3	4.0 (3)	C7—O1—C8—C1	1.22 (18)
C7—C2—C3—C4	0.9 (2)	C7—O1—C8—C9	−178.58 (15)
C1—C2—C3—C4	179.35 (18)	O2—S1—C10—C11	−67.30 (14)
C2—C3—C4—C5	−0.3 (3)	C1—S1—C10—C11	−178.13 (13)
C2—C3—C4—Cl1	179.95 (12)	O2—S1—C10—C14	175.72 (12)
C3—C4—C5—C6	−0.7 (3)	C1—S1—C10—C14	64.89 (14)
Cl1—C4—C5—C6	179.13 (13)	C14—C10—C11—C12	−33.7 (2)
C4—C5—C6—C7	0.9 (3)	S1—C10—C11—C12	−154.09 (13)
C8—O1—C7—C6	178.72 (16)	C10—C11—C12—C13	41.2 (2)
C8—O1—C7—C2	−0.52 (18)	C11—C12—C13—C14	−33.2 (2)
C5—C6—C7—O1	−179.37 (15)	C12—C13—C14—C10	12.1 (2)
C5—C6—C7—C2	−0.2 (3)	C11—C10—C14—C13	13.5 (2)
C3—C2—C7—O1	178.56 (14)	S1—C10—C14—C13	134.01 (15)

### Hydrogen-bond geometry (Å, °)

**Table:**

*D*—H···*A*	*D*—H	H···*A*	*D*···*A*	*D*—H···*A*
C5—H5···O2^i^	0.95	2.61	3.349 (2)	135

Symmetry codes: (i) *x*, *y*−1, *z*.
